# A Comprehensive Simulation Study to Evaluate the Effect Size and Study Length Relationship in Single-Group Interrupted Time Series Analysis

**DOI:** 10.1177/01632787251361514

**Published:** 2025-07-15

**Authors:** Ariel Linden

**Affiliations:** 1 Department of Medicine, Division of Clinical Informatics & Digital Transformation (DoC-IT), 8785 University of California, San Francisco, CA, USA

**Keywords:** interrupted time series analysis, time series regression, sample size, effect size, power, autocorrelation

## Abstract

Single-group interrupted time-series analysis (ITSA) is a popular non-experimental study design in healthcare research. However, little guidance is available to inform the power requirements of ITSA studies under most common usages. We performed simulations to estimate the number of time periods (ranging from 10 to 100) required for percentage increases in level and trend (from baseline), to achieve statistical significance (*p* < 0.05, *p* < 0.01) with >80% and >90% power, when autocorrelation ranges from −0.90 to 0.90, and the intervention is introduced at 33%, 50% and 67% of the time series. Larger effect sizes were required for shorter studies, as well as with increasing autocorrelation, and when the intervention was introduced earlier or later than halfway in the time series. The required effect sizes were generally lower for estimating a change in the level of the time series as compared with the change in the trend, but the opposite was true when the number of time periods was larger. Simulations of studies with 10 time periods consistently produced unreliable estimates. The tables created from these analyses as well as a new community-contributed Stata package called *POWER_ITSA* will guide healthcare researchers in determining the most efficient way to achieve anticipated treatment effects in single-group ITSA studies.

## Introduction

Single-group interrupted time-series analysis (ITSA) is a popular evaluation methodology in healthcare research for non-experimental data in which a single unit (e.g., a patient, a hospital, a county) is observed over time, the dependent variable is a serially ordered outcome at the aggregate level (e.g. morbidity or mortality rates, average costs), and multiple measurements are obtained for both the pre- and post-intervention periods (Linden, 2015). The general study design is called an *interrupted time series* because the intervention is expected to “interrupt” the level and/or trend of the time series, at some point subsequent to its introduction (Campbell & Stanley, 1966). ITSA has been argued to have strong internal validity, primarily through its control over *regression to the mean* (Campbell & Stanley, 1966; Linden, 2007; Shadish et al., 2002), and generally has good external validity, particularly when the unit of measure is at the population level, or when the results can be generalized to other units, treatments or settings (Shadish et al., 2002).

ITSA is used in many areas of healthcare research, with a few recent examples including the effects of clinical guidelines (Bohnert et al., 2018; Bytnar et al., 2021; Marincowitz et al., 2019), interventions that impact health services utilization and cost (Holmgren 2023; Turri et al., 2022; Willer et al., 2024), medication prescribing policies (Chua et al., 2024; Coe et al., 2025; Roberts et al., 2024), healthcare reform (Anderson et al., 2024; Giannouchos et al., 2021; Liu et al., 2020), interventions to improve laboratory testing (Mathura et al., 2021; Sandiford et al., 2018) and community-based interventions (Hilton et al., 2024; Mark et al., 2024), among countless others. ITSA has also been proposed as a more flexible and rapid design to be considered in health research before defaulting to the traditional two-arm randomized controlled trial (Riley 2013), and the Cochrane collaborative, which conducts systematic reviews of the health literature, has recently upgraded its recommendation to now include studies in reviews that used ITSA as the primary research design (EPOC, 2017). Finally, as expected, there has been an explosion of studies in which ITSA has been used as a natural experiment to assess the impact of COVID-19 on a large array of outcomes (a Google Scholar search conducted on March 1, 2025 using the term “COVID-19 interrupted time series analysis” elicited over 129,000 results).

Despite the ubiquity of ITSA studies in healthcare, there appears to be great heterogeneity in how these studies are designed and evaluated (Polus et al., 2017; Turner et al., 2021), with many studies found to be underpowered (Ramsay et al., 2003). The statistical models used in evaluating ITSA studies are complex and, as such, no simple closed-form solution currently exists to compute the sample size requirement. It is therefore of no surprise that several authorities have offered disparate practical recommendations for the minimum sample size needed to achieve sufficient power in ITSA studies. The Effective Practice and Organization of Care (EPOC) Cochrane Group, suggests that ITSA studies have at least 3 time points in each of the pre-intervention and post-intervention periods for inclusion in their reviews (EPOC, 2017). Penfold and Zhang (2013) suggest that a minimum of 8 observations be collected in each of the pre-intervention and post-intervention periods to achieve adequate power, while Ramsay et al. (2003) indicate that 10 pre- and post-intervention data points would elicit at least 80% power to detect a change in the level of five standard deviations (of the pre-intervention data) if the autocorrelation is greater than 0.40.

In contrast to those “rules of thumb” mentioned above, two studies have used simulations to estimate power for a varying number of time periods in ITSA studies. Hawley et al. (2019) generated individual level samples and then aggregated their data for each time point before estimating an ITSA regression model. Their approach differs from the typical “N-of-1” ITSA study where only aggregate data are available. In fact, they found that the underlying sample size per time point had a large impact on power. They also did not account for autocorrelation, rendering these findings of limited utility to investigators that only have access to aggregated and autocorrelated data. Conversely, Zhang et al. (2011) generated simulated data for the “N-of-1” ITSA study type to estimate power at varying numbers of time periods for effect sizes of 0.5, 1.0, and 2.0 where the effect size was defined as the sum of expected level change plus the unit trend change over the standard deviation. However, this effect size metric is unintuitive to most investigators and limiting the simulations to only three effect sizes leaves vast areas of the treatment effects space unmapped. Nonetheless, they found that power increased when sample size or effect size increased, and generally decreased when autocorrelation increased.

This paper contributes to the literature on power for an ITSA study by conducting a comprehensive set of simulations to address factors unique to the basic ITSA design when estimating sample size/power. These factors include the total number of time periods under study, the time point within the overall time series when the intervention is introduced, handling of the autocorrelated nature of the data (i.e., the degree to which errors between consecutive observations are serially correlated), and the measure of effect which can be expressed as either a pre- to post-intervention change in *level* or a pre- to post-intervention change in *trend* of the outcome variable. The emphasis here is to estimate and present effect sizes for the most common levels of power and alpha. More specifically, we ask “what is the required number of treatment periods (sample size) for a given percent change in the level or trend of the time series (effect size) and level of autocorrelation, to achieve statistical significance (*p* < 0.05 or *p* < 0.01) with 80% (or 90%) power?” To achieve this goal, we examine the percent change in level and trend of the time series from baseline using ordinary least squares (OLS) regression adjusted with Newey-West standard errors (Newey & West, 1987). Simulations are repeated for the number of time periods ranging from 10 to 100, autocorrelation ranging from −0.90 to 0.90, and for the introduction of the intervention occurring at one-third, one-half, and two-thirds of the length of the time series. Additionally, a worked example is presented to demonstrate how the simulation approach described in the paper can be used to estimate the number time periods necessary using specific input criteria. The tables created from these analyses, as well as a new community-contributed package for Stata called *POWER_ITSA* will guide healthcare researchers in determining the most efficient way to achieve anticipated treatment effects.

## Methods

### Data Generating Process (DGP)

For each iteration of each simulation, an artificial time series was generated using the Stata^TM^ community-contributed program ITSADGP (Linden, 2025). ITSADGP takes values representing coefficients from the standard ITSA regression model as inputs (Linden, 2015):

(1) $$Y_{t}=\beta_{0}+\beta_{1}T_{t}+\beta_{2}X_{t}+\beta_{3}X_{t}T_{t}+\epsilon_{t}$$

where *Y*_*t*_ is the aggregated outcome variable measured at each equally spaced time point *t*, *T*_*t*_ is the time since the start of the study (commencing at *t* = 0), *X*_*t*_ is a dummy (indicator) variable representing the intervention (pre-intervention periods 0, otherwise 1), and *X*_*t*_*T*_*t*_ is an interaction term between *X*_*t*_ and a sequentially numbered variable starting in the period immediately following the intervention. In the case of a single-group study, *β*_0_ represents the intercept or starting level of the outcome variable. *β*_1_ is the slope or trend of the outcome variable until the introduction of the intervention. *β*_2_ represents the change in the level of the outcome that occurs in the period immediately following the introduction of the intervention from the counterfactual level at that time-point. *β*_3_ represents the difference between pre-intervention and post-intervention slopes of the outcome. Thus, we look for significant *p*-values in *β*_2_ to indicate an immediate treatment effect, or in *β*_3_ to indicate a treatment effect over time (Linden, 2015, 2017).

Additionally, ITSADGP adjusts the time-series for autocorrelation when specified. When the random error terms follow a first-order autoregressive (AR1) process,

(2) $$\epsilon_{t}={\rho \epsilon }_{t-1}+u_{t}$$

where, the autocorrelation parameter *ρ* is the correlation coefficient between adjacent error terms, such that *|ρ| <* 1, and the disturbances *u*_*t*_ are independent *N* (0,*σ*^2^) (see Kutner et al. (2005) for a detailed discussion of autocorrelation in time-series regression models).

**Figure 1.**
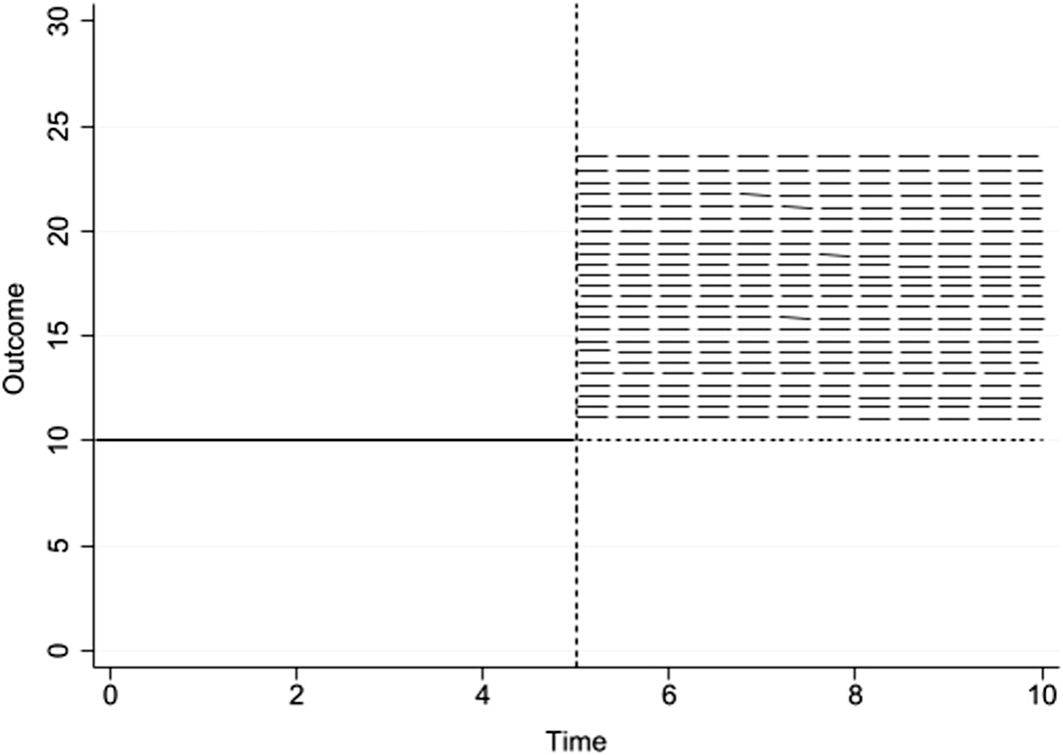
Depiction of the Data Generating Process to Detect a Percent Change in Level Versus the Counterfactual (Baseline Carried Forward, Represented as a Dotted Line)

**Figure 2.**
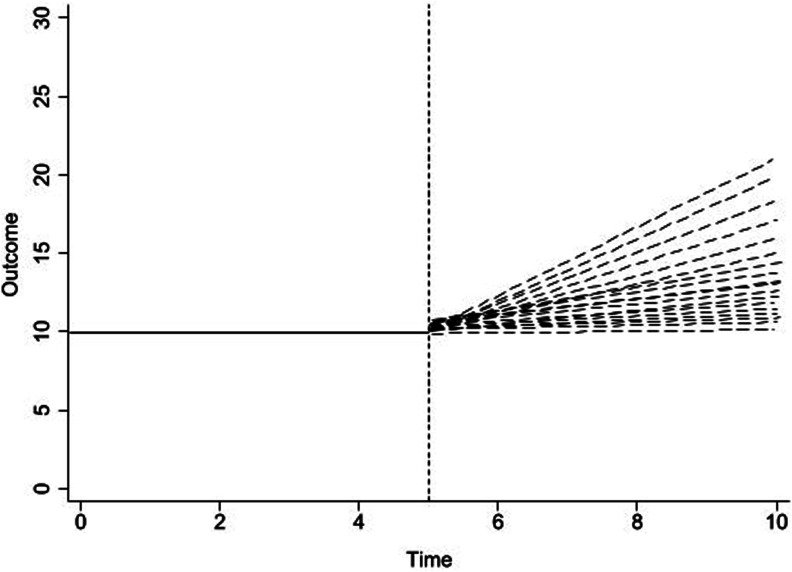
The Data Generating Process to Detect a Percent Change in the Post-intervention Trend From the Pre-intervention Trend

In the current study, the following options were specified as inputs for ITSADGP: the number of time periods in the series (ranging from 10 to 100); the time period when the intervention begins (set at 1/3, 1/2, and 2/3 of the length of the time series); the starting value (intercept) of the time series (set to 10); the trend (slope) of the time series prior to the intervention (set to 0); the change in the level of the time series immediately following the introduction of the intervention versus the counterfactual at that time-point (set to 0 for simulations of change in trend, and set to increase in 5% increments, up to 350% from baseline level, for simulations of level change); the trend (slope) of the time series after introduction of the intervention (set to 0 for change in level, and set to 5% increments up to 350% from baseline trend, for simulations of trend change); the correlation coefficient between adjacent (autoregressive) error terms (ranging from −0.90 to 0.90) and 1 standard deviation used in generating the normally distributed random error term. Figures 1 and 2 visually summarize the DGP for evaluating a percent change in level and trend, respectively.

### Model Estimation

For each individual time series generated, the Stata^TM^ community-contributed package ITSA (Linden, 2015) was employed to estimate the treatment effects of the ITSA represented by coefficients *β*_2_ and *β*_3_ in equation (1) (i.e. the change in level and change in trend, respectively).

This study used the default estimation model implemented in the ITSA package which is a generalized linear model (GLM) adjusting for autocorrelation with Newey-West standard errors (Newey & West, 1987). After model estimation, a Wald test was utilized to determine if the coefficient *β*_2_ or *β*_3_ is 0 (representing the change in level and change in trend, respectively), and the associated *p*-value was saved.

### Simulation

For all scenarios (i.e. varying numbers of time periods, varying levels of autocorrelation, varying the percent change of either level or trend from baseline, and varying the time-point of introduction of the intervention within the time-series) 10,000 simulated datasets were generated and power was computed as the proportion of simulations in which *p* < 0.05 and *p* < 0.01. Finally, the effect sizes (percent increase in level or trend from baseline) corresponding to a power of 80% and 90% (for *p* < 0.05 and *p* < 0.01) were located via a grid search of the simulation output and the values were stored for reporting. Stata^TM^ version 18.0 was used for conducting all analyses.

### Stata Package

While all of the simulations were conducted as described in the sections above to allow for parallel processing, a new community-contributed package for Stata called *POWER_ITSA* (Linden, 2025) allows researchers to easily replicate the methods employed here as well as to conduct power analyses using other criteria as well, such as those described in the Example Section below.

## Results

### Change in Trend

Tables 1–3 present the minimum percent increase in post-intervention trend (vs. baseline trend) needed to achieve >80% power at *p* < 0.05 for an autoregressive (AR1) error model, when the treatment starts at 50%, 33%, and 67% of the time series, respectively. As expected, a greater effect size is required as the number of time periods decreases for the given power and *p*-value. For example, with an autocorrelation coefficient of 0.50, a 50% increase in the post-intervention trend from baseline requires 20 time periods to achieve 80% power at *p* < 0.05, whereas a 15% increase in the post-intervention trend requires 60 time periods (Table 1). Additionally, greater effect sizes are required with increasing autocorrelation. For example, for a study with 30 time periods and autocorrelation of −0.9, a 20% increase in the post-intervention trend is needed to achieve 80% power at *p* < 0.05, whereas a percent increase in trend of 65% is required for the same number of time periods in the study if the autocorrelation is 0.90 (Table 1). The only scenario where this is not true is in the simulated studies with 10 time periods, where greater effect sizes are generally required with decreasing autocorrelation. Moreover, studies with 10 time periods require substantially greater effect sizes than studies with 20 time periods to achieve the desired power and *p* value. These issues are likely explained by the random variability of simulation results for small sample sizes (Zhang et al., 2011).

**Table 1.** Minimum Percent Increase in Post-intervention Trend to Achieve >80% Power at *p* < 0.05 (Treatment Starts at 50% of the Time Series)

	Number of time periods									
rho	10	20	30	40	50	60	70	80	90	100
−0.9	135	35	20	15	10	10	5	5	5	5
−0.8	120	35	20	15	10	10	5	5	5	5
−0.7	115	35	20	15	10	10	5	5	5	5
−0.6	105	35	20	15	10	10	5	5	5	5
−0.5	100	35	20	15	10	10	10	5	5	5
−0.4	100	35	20	15	10	10	10	5	5	5
−0.3	100	40	20	15	10	10	10	5	5	5
−0.2	100	40	20	15	10	10	10	5	5	5
−0.1	100	40	25	15	15	10	10	5	5	5
0.0	100	40	25	15	15	10	10	10	5	5
0.1	100	45	25	20	15	10	10	10	5	5
0.2	100	45	25	20	15	10	10	10	5	5
0.3	110	45	30	20	15	10	10	10	10	5
0.4	110	50	30	20	15	15	10	10	10	10
0.5	110	50	30	20	15	15	10	10	10	10
0.6	110	55	35	25	20	15	15	10	10	10
0.7	110	60	40	25	20	20	15	15	10	10
0.8	110	60	40	30	25	20	20	15	15	10
0.9	110	65	45	35	30	25	20	20	15	15

**Table 2.** Minimum Percent Increase in Post-intervention Trend to Achieve >80% Power at *p* < 0.05 (Treatment Starts at 33% of the Time Series)

	Number of time periods									
rho	10	20	30	40	50	60	70	80	90	100
−0.9	150	55	30	20	15	10	10	10	5	5
−0.8	150	55	30	20	15	10	10	10	5	5
−0.7	150	55	30	20	15	10	10	10	5	5
−0.6	150	55	30	20	15	10	10	10	5	5
−0.5	145	55	30	20	15	10	10	10	10	5
−0.4	140	55	35	20	15	15	10	10	10	5
−0.3	140	55	35	20	15	15	10	10	10	5
−0.2	140	55	35	20	15	15	10	10	10	5
−0.1	135	55	35	20	15	15	10	10	10	10
0.0	135	55	35	25	15	15	10	10	10	10
0.1	135	60	35	25	20	15	10	10	10	10
0.2	135	60	35	25	20	15	10	10	10	10
0.3	135	60	35	25	20	15	15	10	10	10
0.4	135	65	40	25	20	15	15	10	10	10
0.5	130	65	40	25	20	20	15	15	10	10
0.6	130	70	45	30	25	20	15	15	10	10
0.7	130	70	45	35	25	20	15	15	15	10
0.8	130	70	50	40	30	25	20	20	15	15
0.9	130	70	55	40	35	30	25	20	20	20

**Table 3.** Minimum Percent Increase in Post-intervention Trend to Achieve >80% Power at *p* < 0.05 (Treatment Starts at 67% of the Time Series)

	Number of time periods									
rho	10	20	30	40	50	60	70	80	90	100
−0.9	205	55	30	20	15	10	10	10	5	5
−0.8	205	55	30	20	15	10	10	10	5	5
−0.7	200	55	30	20	15	10	10	10	5	5
−0.6	175	55	30	20	15	10	10	10	5	5
−0.5	170	55	30	20	15	10	10	10	10	5
−0.4	160	55	30	20	15	10	10	10	10	5
−0.3	155	55	30	20	15	15	10	10	10	5
−0.2	145	55	35	20	15	15	10	10	10	5
−0.1	140	55	35	20	15	15	10	10	10	5
0.0	135	55	35	20	15	15	10	10	10	10
0.1	130	60	35	25	20	15	10	10	10	10
0.2	130	60	35	25	20	15	10	10	10	10
0.3	130	60	40	25	20	15	15	10	10	10
0.4	130	65	40	25	20	15	15	10	10	10
0.5	130	65	45	30	20	20	15	15	10	10
0.6	130	65	45	30	25	20	15	15	15	10
0.7	130	65	50	35	30	25	20	15	15	10
0.8	130	70	50	35	30	25	25	20	15	15
0.9	130	70	55	40	35	30	25	25	20	20

Also as expected, a somewhat smaller effect size is required to achieve the desired power for studies with an even number of pre- and post-intervention time periods (Table 1) than for studies where the intervention is introduced earlier (Table 2) or later (Table 3) in the time series. However, studies with an intervention introduced in the first-third of the time series (Table 2) has comparable effect size requirements at any given level of autocorrelation and number of time periods to studies where the intervention was introduced at the two-thirds point in the time series (Table 3).

Appendix Tables 1–3, 7–9, 13–15 provide additional effect size estimates for increasing trend needed to achieve >80% and >90% power at *p* < 0.01 and *p* < 0.05. Overall, the findings and interpretation are comparable to those described for Tables 1–3.

### Change in Level

Tables 4–6 present the minimum percent increase in post-intervention level in the period immediately following the intervention (vs. the counterfactual) needed to achieve >80% power at *p* < 0.05 for an autoregressive (AR1) error model, when the treatment starts at 50%, 33%, and 67% of the time series, respectively. As expected, greater effect sizes are needed as the number of time periods decreases and as autocorrelation increases. However, effect size requirements do not appear to differ based on whether the intervention is introduced in the first third, half-way, or two-thirds of the time series. This finding is consistent with that reported in Hawley et al. (2019).

**Table 4.** Minimum Percent Increase in Post-intervention Level to Achieve >80% Power at *p* < 0.05 (Treatment Starts at 50% of the Time Series)

	Number of time periods									
rho	10	20	30	40	50	60	70	80	90	100
−0.9	50	25	20	15	15	15	15	10	10	10
−0.8	45	25	20	15	15	15	15	10	10	10
−0.7	40	25	20	15	15	15	15	10	10	10
−0.6	35	25	20	15	15	15	15	10	10	10
−0.5	35	25	20	15	15	15	15	10	10	10
−0.4	35	25	20	15	15	15	15	15	10	10
−0.3	35	25	20	15	15	15	15	15	10	10
−0.2	35	25	20	20	15	15	15	15	15	10
−0.1	35	25	20	20	15	15	15	15	15	15
0.0	30	25	20	20	20	15	15	15	15	15
0.1	30	25	20	20	20	15	15	15	15	15
0.2	30	25	25	20	20	20	15	15	15	15
0.3	30	25	25	20	20	20	20	15	15	15
0.4	30	30	25	25	25	20	20	20	20	15
0.5	30	30	25	25	25	25	20	20	20	20
0.6	30	30	30	30	25	25	25	20	20	20
0.7	30	30	30	30	30	30	25	25	25	25
0.8	30	35	35	35	30	30	30	30	30	30
0.9	30	35	35	35	35	35	35	35	35	35

**Table 5.** Minimum Percent Increase in Post-intervention Level to Achieve >80% Power at *p* < 0.05 (Treatment Starts at 33% of the Time Series)

	Number of time periods									
rho	10	20	30	40	50	60	70	80	90	100
−0.9	45	25	20	20	15	15	15	15	15	10
−0.8	40	25	20	20	15	15	15	15	15	10
−0.7	40	25	20	20	15	15	15	15	15	10
−0.6	40	25	20	20	15	15	15	15	15	10
−0.5	40	25	20	20	15	15	15	15	15	10
−0.4	35	25	20	20	15	15	15	15	15	10
−0.3	35	25	20	20	15	15	15	15	15	15
−0.2	35	25	20	20	20	15	15	15	15	15
−0.1	35	25	25	20	20	15	15	15	15	15
0.0	35	25	25	20	20	15	15	15	15	15
0.1	35	25	25	20	20	20	15	15	15	15
0.2	35	30	25	20	20	20	20	15	15	15
0.3	35	30	25	25	20	20	20	20	15	15
0.4	30	30	25	25	25	20	20	20	20	20
0.5	30	30	25	25	25	25	20	20	20	20
0.6	30	30	30	30	25	25	25	25	25	20
0.7	30	30	30	30	30	30	30	25	25	25
0.8	30	30	35	35	35	35	30	30	30	30
0.9	30	35	35	35	35	35	35	35	35	35

**Table 6.** Minimum Percent Increase in Post-intervention Level to Achieve >80% Power at *p* < 0.05 (Treatment Starts at 67% of the Time Series)

	Number of time periods									
rho	10	20	30	40	50	60	70	80	90	100
−0.9	55	25	20	20	15	15	15	15	10	10
−0.8	45	25	20	20	15	15	15	15	10	10
−0.7	35	25	20	20	15	15	15	15	10	10
−0.6	35	25	20	20	15	15	15	15	10	10
−0.5	35	25	20	20	15	15	15	15	15	10
−0.4	30	25	20	20	15	15	15	15	15	10
−0.3	30	25	20	20	15	15	15	15	15	10
−0.2	30	25	20	20	15	15	15	15	15	15
−0.1	30	25	20	20	20	15	15	15	15	15
0.0	30	25	20	20	20	15	15	15	15	15
0.1	30	25	25	20	20	20	15	15	15	15
0.2	30	25	25	20	20	20	15	15	15	15
0.3	30	25	25	25	20	20	20	20	15	15
0.4	30	25	25	25	20	20	20	20	20	20
0.5	30	30	25	25	25	20	20	20	20	20
0.6	30	30	30	25	25	25	25	25	20	20
0.7	30	30	30	30	30	30	25	25	25	25
0.8	30	30	30	35	30	30	30	30	30	30
0.9	30	30	35	35	35	35	35	35	35	35

Of note, effect sizes needed to detect a statistical change in the level of the time series are generally lower than those needed to detect a statistical change in the trend of the time series when the number of time periods in the study are small, while the converse is true when the number of time periods in the study is large. For example, a minimum increase of 30% in the level of the time series is required if there are 20 time periods, and an autocorrelation of 0.50 to achieve 80% power at *p* < 0.05 (Table 4) whereas a minimum increase of 50% in the trend is required for the same criteria (Table 1). Conversely, if there are 50 time periods in the study and an autocorrelation coefficient of 0.50, a minimum change of 25% in the level is required to achieve 80% power at *p* < 0.05 (Table 4), whereas a minimum change of only 15% in the trend is required to achieve 80% power at *p* < 0.05 (Table 1).

Appendix Tables 4–6, 10–12, 16–18 provide additional effect size estimates for percent increases in level needed to achieve >80% and >90% power at *p* < 0.01 and *p* < 0.05. Overall, the findings and interpretation are comparable to those described for Tables 4–6.

## Example

In this section we provide a worked example to demonstrate how the simulation approach described herein can be tailored for a specific research study, where the investigator wants to determine the number of time periods necessary for a given effect size to achieve statistical significance.

A small medical practice was about to implement an artificial intelligence (AI) based transcription program. During an office visit, the program would listen to the conversation between clinician and patient and write up the medical documentation. The hypothesis was that by freeing up the clinician from performing this activity, office visits would become more comprehensive when necessary, and/or more office visits could be scheduled over the course of a work-week. In turn, this would lead to an increase in the number of billing units (reflecting the greater effort and time involved in patient care) that the medical group could submit for reimbursement from payors.

The medical group wanted to estimate how many weeks of implementation it would take for the program to be deemed “successful”, where success was defined as a statistically significant increase in the number of weekly billing units. The assumptions (model inputs) were as follows: the baseline level (intercept) of billing units was 500 per week; the pre-intervention trend was 0 (given that no other unique event or secular trend occurred up until implementation); the change in level was set to 0 (because it was hypothesized that it would take time for efficiencies to be realized from the program); the post-intervention trend was set to 0.20 (with the assumption being that there would be 20% weekly increase in trend of billing units after implementation; and autocorrelation was set to 0.20 (based on analysis of past weekly billing units). 10,000 datasets were simulated with the number of weekly time periods ranging from 10 to 60 in increments of 1, with a balanced number of pre- and post-intervention time periods.

The results indicated that it would take 34 total weeks (17 weeks of implementation) to achieve a statistically significant (*p* < 0.05) treatment effect (20% increase in weekly trend) with 80% power. This result is comparable to that found in Table 1 (when cross-referencing an autocorrelation of 0.20, we see that it would take between 30 and 40 time periods to achieve an effect size between 20% to 25%). The estimated number of time periods required to achieve statistical significance (*p* < 0.05) with 90% power was computed to be 39 weeks (20 weeks of implementation), which is nearly identical to the 40 time periods presented in Appendix Table 4 for the same power (90%) and alpha level (*p* < 0.05) criteria.

## Discussion

Given the applicability of ITSA to address a tremendous array of research questions, healthcare investigators need guidance in designing studies for which ITSA will be the evaluation approach. With the emphasis on the number of time periods required for a minimum effect size to achieve the desired power for a given level of autocorrelation, the results of the simulations reported in this paper highlight several factors that a healthcare researcher must consider when determining the most efficient way to conduct an ITSA study.

One issue to consider is the relationship between treatment effect size and sample size. In the common “N-of-1” ITSA design, sample size refers to the number of time periods in which data are collected, and not the underlying number of study participants, as in other study designs. Therefore, a healthcare researcher planning to utilize an ITSA design for evaluation must anticipate the study duration required to achieve the expected treatment effect. This has implications for both funding and data collection. For example, Table 1 shows that 100 time periods are necessary for a 5% increase in the time series trend to achieve statistical significance (*p* < 0.05) with 80% power. If these data represent monthly observations, then it would take somewhere between 5.8 and 8.3 years (depending on the expected autocorrelation) for that 5% change in trend to achieve statistical significance (*p* < 0.05). It may be difficult to receive funding for a study of that duration, and data collection sources and methods may change or even cease to be available over such a lengthy period of time. Naturally, if time is a constraint, the emphasis should be on maximizing the treatment effect over a shorter duration.

Another issue to consider (and related to the first), is the measure that will be used to determine the intervention’s effect – a change in level, or a change in trend. As the simulation results show, for shorter duration studies, the required effect sizes are generally lower for estimating a change in the level of the time series as compared with the change in the trend. The opposite is true when the number of time periods in the study is larger. This is likely explained by how the parameters for a change in level and change in trend are estimated in an ITSA model (Equation (1)). The change in level (*β*_2_) represents an abrupt shift of the time series in the period immediately following introduction of the intervention, resulting in a more easily detected signal when comparing against the pre-intervention level (*β*_0_). Conversely, the change in trend (*β*_3_) represents the difference between the average slope of all the post-intervention periods (*β*_1_ + *β*_3_) and the average slope of all the pre-intervention periods (*β*_1_) (Linden, 2015, 2017). Consequently, the change in trend is typically more subtle than the change in level, requiring more time periods to detect a signal. Taken together, this suggests that level changes are more readily detected in a study with a smaller number of time periods, and changes in trend are more likely detected as the number of time periods increase. Therefore, a healthcare researcher should anticipate the type of effect the intervention will experience when considering which effect measure to use in estimating power. For example, assume an intervention involves creating a prompt in the electronic health record that will *require* physicians to perform a specific task when treating all new patients. This will likely result in a large, immediate change in the time series of the outcome under study, because physicians must perform the task. In this case, the investigator should estimate the number of time periods required for the “change in level” effect to achieve statistical significance. Now assume that the prompt only *suggests* that physicians perform a specific task when treating all new patients. If there is an effect, it will likely be small and take a long time for that effect to achieve statistical significance, because some physicians may not believe that the task is conducive to improving the desired outcome. In this case, the healthcare researcher should estimate the number of time periods required for the “change in trend” effect to achieve statistical significance, while ensuring that the intervention is sufficiently robust to attain the anticipated treatment effect (perhaps by including a physician training component). Based on the simulations conducted here, it appears that 20 time periods of observation is roughly the transition when it becomes easier to detect a change in trend from a change in level.

From a statistical perspective, controlling for the effects of autocorrelation is crucial. As the simulation results show, greater effect sizes are required with increasing autocorrelation, holding the number of time periods and power constant. Stated differently, with increasing autocorrelation comes lower power, holding effect size and the number of time periods constant (as reported in Zhang et al., 2011). One may question why autocorrelation still negatively impacts power even though the regression model used in the simulations was designed to control for serial correlation (i.e. time series regression with Newey-West standard errors)? The simple answer is that even when estimating effects using a time series regression model, the presence of autocorrelation may still introduce model inefficiency, potential heteroscedasticity, and possibly model misspecification, all of which can reduce the statistical power. In other words, these models can behave poorly when there is substantial autocorrelation, especially when the sample size is small (where small can be even as large as 100 time points (Wooldridge, 2016)). Wooldridge (2016) suggests methods to improve upon the adjustment for autocorrelation in time series regression models, however it is unclear how the results of these actions may affect estimates in ITSA designs. Given that only some of the lost power can be recovered through the use of time series regression, the most practical advice may be to “over-correct” for the expected level of autocorrelation when planning the study. For example, if the required percent increase in the trend for an autocorrelation of 0.50 is 30% for a study with 30 time periods, the researcher may increase the expected autocorrelation to 0.60 or 0.70, which raises the required effect size to 35% or 40%, respectively. Alternatively, one can simply apply an autocorrelation ranging from 0.10 to 0.50, as suggested by Zhang et al. (2011).

Finally, the simulation results reported here indicate that studies with only 10 time periods produce unreliable estimates. This is manifested by effect sizes that are up to four times greater than those of studies with 20 time periods, and autocorrelation effects that are in near reverse order to those obtained for any other sample size. These findings are consistent with those reported by Zhang et al. (2011) for studies with 12 time periods. Thus, it is fair to conclude that investigators should be cautious when interpreting results of ITSA studies with fewer than 20 time periods, lest their statistically significant result be a function of a type I error when using the specific modeling approach implemented here (time-series regression with Newey-West standard errors on a continuous outcome). One possible solution to this concern is to increase the frequency of data collection over the existing study duration. For example, a study with a 12-month duration would benefit, statistically, from collecting data on a weekly or bi-weekly basis, assuming that the outcome time-series is trending in a consistent direction. However, reporting more observations over an overall shorter duration may not be considered as “clinically” meaningful as fewer observations collected over an overall longer duration. This is something that an investigator will have to consider, relying on content expertise and following accepted practices in their discipline.

While the current paper includes Tables for investigators that are based on general guidance related to the various components of power and their interactions, the simulation methods implemented here can be tailored to use specific inputs, much in the same way that empirically-driven power calculations are used for generating estimates. This was demonstrated in the worked example. The community-contributed Stata package *POWER_ITSA* (Linden, 2025) allows researchers to replicate the results of the simulation study and the worked example, as well as design their own power analyses.

The present study has limitations. The simulation strategy developed here was designed to replicate the most common ITSA study in healthcare research: a single-group (“N-of-1”) with a single treatment (intervention) period, in which time series regression is used as the statistical model and assumes a first-order autocorrelation (lag = 1) and treats the outcome variable as continuous (regardless of true data type). This implies that the several factors were not included in the simulation design: multiple consecutive interventions, seasonality, higher-order autocorrelation, and different outcome variable types. Each one of these elements adds further complexity to an ITSA study, which likely explains why they are rarely considered in practice. Nonetheless, future research should investigate how power in ITSA studies is affected by these factors. Also, the data generating process implemented in the simulations may not adequately represent all scenarios found in empirical research. Nonetheless, the results reported here are qualitatively comparable to those reported by Zhang et al. (2011) using a different data generating process, adding confidence that the results may be generalizable.

Finally, it is important to note that while the single-group ITSA is the obvious design choice when no control group is available (such as when an intervention is implemented across all study units simultaneously or at the population level), a multiple-group ITSA is always the preferred design when one or more comparable control groups are available for comparison to improve causal inference (Linden, 2018a, 2018b). Future research should extend the analyses conducted herein to investigate the effect size and study length relationship for the multiple-group ITSA design.

## Conclusion

Based on the results of a comprehensive set of simulations, this paper provides guidance on estimating sample size/power in single-group ITSA studies for which time series regression will be used as the statistical model. Healthcare researchers must consider the many factors highlighted here that affect sample size/power when determining the most efficient way to conduct an ITSA study.

## Supplemental Material

Supplemental Material - A Comprehensive Simulation Study to Evaluate the Effect Size and Study Length Relationship in Single-Group Interrupted Time Series Analysis Supplemental Material for A Comprehensive Simulation Study to Evaluate the Effect Size and Study Length Relationship in Single-Group Interrupted Time Series Analysis by Ariel Linden in Evaluation & the Health Professions
