# Supplementary material for: A Comprehensive Simulation Study to Evaluate the Effect Size and Study Length Relationship in Single-Group Interrupted Time Series Analysis
Source: Eval Health Prof. 2025 Jul 15;49(2):181–90. doi: 10.1177/01632787251361514 (PMC13069133; doi:10.1177/01632787251361514)
Supplement: Supplemental Material - A Comprehensive Simulation Study to Evaluate the Effect Size and Study Length Relationship in Single-Group Interrupted Time Series Analysis [file sj-pdf-1-ehp-10.1177_01632787251361514.pdf]

Supplemental Material

Appendix Table 1

Minimum percent increase in post-intervention trend to achieve >90% power at $p<0.05$ (treatment starts at 50% of the time series).

	Number of time periods									
rho	10	20	30	40	50	60	70	80	90	100
-0.9	175	45	25	15	10	10	10	5	5	5
-0.8	145	45	25	15	10	10	10	5	5	5
-0.7	135	45	25	15	10	10	10	5	5	5
-0.6	125	45	25	15	10	10	10	5	5	5
-0.5	120	45	25	15	10	10	10	5	5	5
-0.4	120	45	25	15	15	10	10	5	5	5
-0.3	120	45	25	15	15	10	10	10	5	5
-0.2	120	45	25	20	15	10	10	10	5	5
-0.1	120	45	25	20	15	10	10	10	5	5
0.0	120	50	30	20	15	10	10	10	10	5
0.1	120	50	30	20	15	15	10	10	10	5
0.2	130	50	30	20	15	15	10	10	10	10
0.3	130	55	30	25	20	15	10	10	10	10
0.4	130	60	35	25	20	15	15	10	10	10
0.5	140	60	40	30	20	15	15	10	10	10
0.6	140	70	45	30	25	20	15	15	10	10
0.7	145	80	50	35	25	20	20	15	15	10
0.8	150	80	55	40	30	25	25	20	15	15
0.9	150	90	60	45	35	30	30	25	20	20

Appendix Table 2

Minimum percent increase in post-intervention trend to achieve >90% power at $p<0.05$ (treatment starts at 33% of the time series).

	Number of time periods									
rho	10	20	30	40	50	60	70	80	90	100
-0.9	220	65	40	20	15	15	10	10	10	5
-0.8	200	65	40	20	15	15	10	10	10	5
-0.7	200	65	40	20	15	15	10	10	10	5
-0.6	185	65	40	25	15	15	10	10	10	5
-0.5	190	65	40	25	15	15	10	10	10	5
-0.4	180	65	40	25	15	15	10	10	10	5
-0.3	190	65	40	25	20	15	10	10	10	10
-0.2	190	65	40	25	20	15	10	10	10	10
-0.1	175	70	40	25	20	15	10	10	10	10
0.0	175	70	40	25	20	15	15	10	10	10
0.1	175	70	40	25	20	15	15	10	10	10
0.2	175	70	45	25	20	20	15	10	10	10
0.3	175	75	45	30	25	20	15	15	10	10
0.4	175	75	50	30	25	20	15	15	10	10
0.5	175	80	50	35	25	20	15	15	15	10
0.6	175	85	55	40	30	25	20	15	15	15
0.7	175	90	60	40	35	25	20	20	15	15
0.8	175	95	65	45	40	30	25	25	20	20
0.9	175	100	70	50	45	35	30	30	25	25

Appendix Table 3

Minimum percent increase in post-intervention trend to achieve >90% power at p<0.05 (treatment starts at 67% of the time series).

	Number of time periods									
rho	10	20	30	40	50	60	70	80	90	100
-0.9	310	65	35	20	15	15	10	10	10	5
-0.8	300	65	35	20	15	15	10	10	10	5
-0.7	275	65	35	20	15	15	10	10	10	5
-0.6	245	65	35	20	15	15	10	10	10	5
-0.5	220	65	35	20	15	15	10	10	10	5
-0.4	205	65	35	25	15	15	10	10	10	10
-0.3	200	65	40	25	20	15	10	10	10	10
-0.2	175	70	40	25	20	15	10	10	10	10
-0.1	170	70	40	25	20	15	15	10	10	10
0.0	170	70	40	25	20	15	15	10	10	10
0.1	170	70	40	25	20	15	15	10	10	10
0.2	165	70	45	30	20	20	15	10	10	10
0.3	165	75	45	30	25	20	15	15	10	10
0.4	165	80	50	35	25	20	15	15	15	10
0.5	165	85	50	35	30	20	20	15	15	10
0.6	165	85	55	40	30	25	20	20	15	15
0.7	160	85	60	45	35	30	25	20	20	15
0.8	160	90	65	50	40	35	30	25	20	20
0.9	160	95	70	55	45	40	35	30	25	25

Appendix Table 4

Minimum percent increase in post-intervention level to achieve >90% power at p<0.05 (treatment starts at 50% of the time series).

	Number of time periods									
rho	10	20	30	40	50	60	70	80	90	100
-0.9	65	30	25	20	15	15	15	15	15	15
-0.8	60	30	25	20	15	15	15	15	15	15
-0.7	50	30	25	20	15	15	15	15	15	15
-0.6	45	30	25	20	15	15	15	15	15	15
-0.5	45	30	25	20	20	15	15	15	15	15
-0.4	40	30	25	20	20	15	15	15	15	15
-0.3	40	30	25	20	20	15	15	15	15	15
-0.2	40	30	25	20	20	15	15	15	15	15
-0.1	40	30	25	20	20	20	15	15	15	15
0.0	40	30	25	20	20	20	15	15	15	15
0.1	40	30	25	25	20	20	20	20	15	15
0.2	40	30	25	25	25	20	20	20	15	15
0.3	40	30	30	25	25	20	20	20	20	20
0.4	40	35	30	30	25	25	25	20	20	20
0.5	40	35	35	30	25	25	25	25	20	20
0.6	40	35	35	35	30	30	30	25	25	25
0.7	40	40	40	35	35	35	30	30	30	30
0.8	40	45	45	40	40	40	35	35	35	35
0.9	40	45	45	45	45	45	40	40	40	40

Appendix Table 5

Minimum percent increase in post-intervention level to achieve >90% power at $p<0.05$ (treatment starts at 33% of the time series).

	Number of time periods									
rho	10	20	30	40	50	60	70	80	90	100
-0.9	60	30	25	20	20	15	15	15	15	15
-0.8	55	30	25	20	20	15	15	15	15	15
-0.7	55	30	25	20	20	15	15	15	15	15
-0.6	50	30	25	20	20	15	15	15	15	15
-0.5	45	30	25	20	20	15	15	15	15	15
-0.4	45	30	25	20	20	15	15	15	15	15
-0.3	45	30	25	20	20	20	15	15	15	15
-0.2	45	30	25	20	20	20	15	15	15	15
-0.1	45	30	25	25	20	20	20	15	15	15
0.0	45	30	25	25	20	20	20	20	15	15
0.1	45	30	30	25	20	20	20	20	20	15
0.2	45	35	30	25	25	20	20	20	20	20
0.3	45	35	30	25	25	25	20	20	20	20
0.4	45	35	30	30	30	25	25	25	20	20
0.5	45	35	35	30	30	30	25	25	25	25
0.6	40	40	35	35	35	30	30	30	25	25
0.7	40	40	40	40	40	35	35	30	30	30
0.8	40	40	45	45	40	40	40	35	35	35
0.9	40	45	45	45	45	45	45	40	40	40

Appendix Table 6

Minimum percent increase in post-intervention level to achieve >90% power at p<0.05 (treatment starts at 67% of the time series).

	Number of time periods									
rho	10	20	30	40	50	60	70	80	90	100
-0.9	70	30	25	20	20	15	15	15	15	15
-0.8	55	30	25	20	20	15	15	15	15	15
-0.7	50	30	25	20	20	15	15	15	15	15
-0.6	45	30	25	20	20	15	15	15	15	15
-0.5	45	30	25	20	20	15	15	15	15	15
-0.4	40	30	25	20	20	15	15	15	15	15
-0.3	35	30	25	20	20	15	15	15	15	15
-0.2	35	30	25	20	20	20	15	15	15	15
-0.1	35	30	25	20	20	20	20	15	15	15
0.0	35	30	25	20	20	20	20	20	15	15
0.1	35	30	25	25	20	20	20	20	20	15
0.2	35	30	25	25	25	20	20	20	20	15
0.3	35	30	30	25	25	20	20	20	20	20
0.4	35	35	30	30	25	25	25	25	20	20
0.5	35	35	30	30	30	25	25	25	25	25
0.6	35	35	35	35	30	30	30	25	25	25
0.7	35	40	35	35	35	35	35	30	30	30
0.8	35	40	40	40	40	40	40	35	35	35
0.9	35	45	45	45	45	45	45	40	40	40

Appendix Table 7

Minimum percent increase in post-intervention trend to achieve >80% power at p<0.01 (treatment starts at 50% of the time series).

	Number of time periods									
rho	10	20	30	40	50	60	70	80	90	100
-0.9	205	65	40	25	20	15	10	10	10	5
-0.8	185	65	40	25	20	15	10	10	10	5
-0.7	185	65	40	25	20	15	10	10	10	5
-0.6	180	65	40	25	20	15	10	10	10	10
-0.5	180	65	40	25	20	15	10	10	10	10
-0.4	180	65	40	25	20	15	10	10	10	10
-0.3	175	65	40	25	20	15	10	10	10	10
-0.2	170	65	40	25	20	15	10	10	10	10
-0.1	165	70	40	25	20	15	10	10	10	10
0.0	160	70	40	25	20	15	15	10	10	10
0.1	160	70	45	25	20	15	15	10	10	10
0.2	160	70	45	30	20	20	15	10	10	10
0.3	160	70	45	30	25	20	15	15	10	10
0.4	155	75	45	30	25	20	15	15	10	10
0.5	155	75	45	35	25	20	15	15	15	10
0.6	150	80	50	35	30	25	20	15	15	15
0.7	150	80	55	40	30	25	20	20	15	15
0.8	150	85	60	45	35	30	25	20	20	15
0.9	150	90	65	50	40	35	30	25	25	20

Appendix Table 8

Minimum percent increase in post-intervention trend to achieve >80% power at p<0.01 (treatment starts at 33% of the time series).

	Number of time periods									
rho	10	20	30	40	50	60	70	80	90	100
-0.9	205	65	40	25	20	15	10	10	10	5
-0.8	185	65	40	25	20	15	10	10	10	5
-0.7	185	65	40	25	20	15	10	10	10	5
-0.6	180	65	40	25	20	15	10	10	10	10
-0.5	180	65	40	25	20	15	10	10	10	10
-0.4	180	65	40	25	20	15	10	10	10	10
-0.3	175	65	40	25	20	15	10	10	10	10
-0.2	170	65	40	25	20	15	10	10	10	10
-0.1	165	70	40	25	20	15	10	10	10	10
0.0	160	70	40	25	20	15	15	10	10	10
0.1	160	70	45	25	20	15	15	10	10	10
0.2	160	70	45	30	20	20	15	10	10	10
0.3	160	70	45	30	25	20	15	15	10	10
0.4	155	75	45	30	25	20	15	15	10	10
0.5	155	75	45	35	25	20	15	15	15	10
0.6	150	80	50	35	30	25	20	15	15	15
0.7	150	80	55	40	30	25	20	20	15	15
0.8	150	85	60	45	35	30	25	20	20	15
0.9	150	90	65	50	40	35	30	25	25	20

Appendix Table 9

Minimum percent increase in post-intervention trend to achieve >80% power at p<0.01 (treatment starts at 67% of the time series).

	Number of time periods									
rho	10	20	30	40	50	60	70	80	90	100
-0.9	310	65	40	25	20	15	15	15	15	10
-0.8	300	65	40	25	20	15	15	15	15	10
-0.7	260	65	40	25	20	15	15	15	15	10
-0.6	230	65	40	25	20	15	15	15	15	10
-0.5	215	65	40	25	20	15	15	15	15	10
-0.4	205	65	40	25	20	15	15	15	15	10
-0.3	185	65	40	25	20	15	15	15	15	10
-0.2	175	70	40	25	20	15	15	15	15	10
-0.1	170	70	40	25	20	15	15	15	15	10
0.0	165	70	40	25	20	15	15	15	15	10
0.1	150	70	40	25	20	15	15	15	15	10
0.2	150	70	45	30	20	20	15	15	15	10
0.3	150	70	45	30	25	20	15	15	15	10
0.4	150	75	45	30	25	20	15	15	15	10
0.5	145	75	50	35	25	20	15	15	15	10
0.6	145	75	50	35	30	25	20	15	15	15
0.7	145	80	55	40	35	25	25	20	15	15
0.8	145	85	55	45	35	30	25	20	20	15
0.9	145	85	60	50	40	35	30	25	25	20

Appendix Table 10

Minimum percent increase in post-intervention level to achieve >80% power at p<0.01 (treatment starts at 50% of the time series).

	Number of time periods									
rho	10	20	30	40	50	60	70	80	90	100
-0.9	65	30	25	20	20	15	15	15	15	15
-0.8	55	30	25	20	20	15	15	15	15	15
-0.7	50	30	25	20	20	15	15	15	15	15
-0.6	45	30	25	20	20	15	15	15	15	15
-0.5	45	30	25	20	20	15	15	15	15	15
-0.4	40	30	25	20	20	15	15	15	15	15
-0.3	40	30	25	20	20	15	15	15	15	15
-0.2	40	30	25	20	20	20	15	15	15	15
-0.1	40	30	25	20	20	20	15	15	15	15
0.0	40	30	25	25	20	20	20	15	15	15
0.1	35	30	25	25	20	20	20	20	15	15
0.2	35	30	30	25	25	20	20	20	20	15
0.3	35	30	30	25	25	20	20	20	20	20
0.4	35	35	30	30	25	25	25	20	20	20
0.5	35	35	30	30	25	25	25	25	20	20
0.6	35	35	35	30	30	30	30	25	25	25
0.7	35	35	35	35	35	30	30	30	30	30
0.8	35	40	40	40	40	35	35	35	35	35
0.9	35	40	40	40	40	40	40	40	40	40

Appendix Table 11

Minimum percent increase in post-intervention level to achieve >80% power at p<0.01 (treatment starts at 33% of the time series).

	Number of time periods									
rho	10	20	30	40	50	60	70	80	90	100
-0.9	55	30	25	20	20	20	15	15	15	15
-0.8	50	30	25	20	20	20	15	15	15	15
-0.7	50	30	25	20	20	20	15	15	15	15
-0.6	45	30	25	20	20	20	15	15	15	15
-0.5	45	30	25	20	20	20	15	15	15	15
-0.4	45	30	25	20	20	20	15	15	15	15
-0.3	45	30	25	25	20	20	20	15	15	15
-0.2	45	30	25	25	20	20	20	20	15	15
-0.1	40	30	25	25	20	20	20	20	15	15
0.0	40	30	25	25	25	20	20	20	15	15
0.1	40	30	30	25	25	20	20	20	20	15
0.2	40	35	30	25	25	20	20	20	20	20
0.3	40	35	30	25	25	25	20	20	20	20
0.4	40	35	30	30	30	25	25	25	20	20
0.5	40	35	35	30	30	25	25	25	25	25
0.6	40	35	35	35	30	30	30	25	25	25
0.7	40	35	35	35	35	35	35	30	30	30
0.8	35	40	40	40	40	40	35	35	35	35
0.9	35	40	40	45	45	45	40	40	40	40

Appendix Table 12

Minimum percent increase in post-intervention level to achieve >80% power at p<0.01 (treatment starts at 67% of the time series).

	Number of time periods									
rho	10	20	30	40	50	60	70	80	90	100
-0.9	70	30	25	20	20	20	15	15	15	15
-0.8	55	30	25	20	20	20	15	15	15	15
-0.7	50	30	25	20	20	20	15	15	15	15
-0.6	45	30	25	20	20	20	15	15	15	15
-0.5	40	30	25	20	20	20	15	15	15	15
-0.4	40	30	25	20	20	20	15	15	15	15
-0.3	35	30	25	20	20	20	15	15	15	15
-0.2	35	30	25	20	20	20	20	15	15	15
-0.1	35	30	25	20	20	20	20	15	15	15
0.0	35	30	25	25	20	20	20	20	15	15
0.1	35	30	25	25	20	20	20	20	20	20
0.2	35	30	25	25	25	20	20	20	20	20
0.3	35	30	30	25	25	25	20	20	20	20
0.4	35	30	30	30	25	25	25	25	20	20
0.5	35	35	30	30	30	25	25	25	25	25
0.6	35	35	35	30	30	30	30	30	25	25
0.7	35	35	35	35	35	35	30	30	30	30
0.8	35	35	35	40	35	35	35	35	35	35
0.9	35	35	40	45	45	45	45	45	45	45

Appendix Table 13

Minimum percent increase in post-intervention trend to achieve >90% power at p<0.01 (treatment starts at 50% of the time series).

	Number of time periods									
rho	10	20	30	40	50	60	70	80	90	100
-0.9	205	50	30	20	15	10	10	10	5	5
-0.8	200	50	30	20	15	10	10	10	5	5
-0.7	160	50	30	20	15	10	10	10	5	5
-0.6	150	50	30	20	15	10	10	10	5	5
-0.5	145	50	30	20	15	10	10	10	5	5
-0.4	145	50	30	20	15	10	10	10	10	5
-0.3	145	55	30	20	15	10	10	10	10	5
-0.2	145	55	30	20	15	10	10	10	10	5
-0.1	145	55	30	20	15	15	10	10	10	5
0.0	145	60	35	20	15	15	10	10	10	10
0.1	145	60	35	25	15	15	10	10	10	10
0.2	150	60	35	25	20	15	15	10	10	10
0.3	155	65	40	25	20	15	15	10	10	10
0.4	155	70	40	30	25	15	15	10	10	10
0.5	155	70	45	30	25	20	15	15	10	10
0.6	160	75	50	35	25	20	20	15	15	10
0.7	160	90	55	40	30	25	20	15	15	15
0.8	165	100	60	45	35	30	25	20	20	15
0.9	165	105	65	50	40	35	30	25	25	20

Appendix Table 14

Minimum percent increase in post-intervention trend to achieve >90% power at p<0.01 (treatment starts at 33% of the time series).

	Number of time periods									
rho	10	20	30	40	50	60	70	80	90	100
-0.9	260	80	45	25	20	15	10	10	10	10
-0.8	245	80	45	25	20	15	10	10	10	10
-0.7	240	80	45	25	20	15	15	10	10	10
-0.6	225	80	45	25	20	15	15	10	10	10
-0.5	225	80	45	25	20	15	15	10	10	10
-0.4	225	80	45	25	20	15	15	10	10	10
-0.3	225	80	45	25	20	15	15	10	10	10
-0.2	220	80	45	30	20	15	15	10	10	10
-0.1	210	80	45	30	25	20	15	10	10	10
0.0	205	80	45	30	25	20	15	15	10	10
0.1	205	85	50	30	25	20	15	15	10	10
0.2	205	85	50	35	25	20	15	15	10	10
0.3	205	85	55	35	25	20	15	15	15	10
0.4	205	90	55	35	30	25	20	15	15	10
0.5	205	95	60	40	30	25	20	15	15	15
0.6	205	100	60	45	35	25	20	20	15	15
0.7	195	105	65	45	35	30	25	20	20	15
0.8	200	110	70	50	40	35	30	25	25	20
0.9	200	115	75	55	45	40	35	30	30	25

Appendix Table 15

Minimum percent increase in post-intervention trend to achieve >90% power at p<0.01 (treatment starts at 67% of the time series).

	Number of time periods									
rho	10	20	30	40	50	60	70	80	90	100
-0.9	310	80	45	25	20	15	15	10	10	10
-0.8	310	80	45	25	20	15	15	10	10	10
-0.7	310	80	45	25	20	15	15	10	10	10
-0.6	300	80	45	25	20	15	15	10	10	10
-0.5	275	80	45	25	20	15	15	10	10	10
-0.4	265	80	45	30	20	15	15	10	10	10
-0.3	265	80	45	30	20	15	15	10	10	10
-0.2	225	80	45	30	20	20	15	10	10	10
-0.1	210	80	45	30	20	20	15	10	10	10
0.0	205	80	45	30	25	20	15	15	10	10
0.1	200	85	50	30	25	20	15	15	10	10
0.2	195	85	50	35	25	20	15	15	10	10
0.3	190	85	55	35	25	20	15	15	15	10
0.4	190	90	55	40	30	25	20	15	15	10
0.5	185	95	60	40	30	25	20	15	15	15
0.6	185	95	65	45	35	30	25	20	15	15
0.7	185	100	65	50	35	30	25	20	20	15
0.8	175	105	70	55	40	35	30	25	25	20
0.9	175	110	75	60	45	40	35	30	30	25

Appendix Table 16

Minimum percent increase in post-intervention level to achieve >90% power at p<0.01 (treatment starts at 50% of the time series).

	Number of time periods									
rho	10	20	30	40	50	60	70	80	90	100
-0.9	80	30	25	25	20	20	20	15	15	15
-0.8	75	30	25	25	20	20	20	15	15	15
-0.7	65	30	25	25	20	20	20	15	15	15
-0.6	55	30	25	25	20	20	20	15	15	15
-0.5	55	30	25	25	20	20	20	15	15	15
-0.4	50	30	25	25	20	20	20	15	15	15
-0.3	50	30	30	25	20	20	20	15	15	15
-0.2	45	30	30	25	20	20	20	20	15	15
-0.1	45	30	30	25	20	20	20	20	20	15
0.0	45	35	30	25	25	20	20	20	20	20
0.1	45	35	30	25	25	25	20	20	20	20
0.2	45	35	30	30	25	25	20	20	20	20
0.3	45	40	35	30	30	25	25	25	20	20
0.4	45	40	35	30	30	30	25	25	25	25
0.5	45	40	40	35	30	30	30	25	25	25
0.6	45	40	40	40	35	35	30	30	30	30
0.7	45	45	45	40	40	40	35	35	35	35
0.8	45	50	50	45	45	45	40	40	40	40
0.9	40	50	50	50	45	45	45	45	45	45

Appendix Table 17

Minimum percent increase in post-intervention level to achieve >90% power at p<0.01 (treatment starts at 33% of the time series).

	Number of time periods									
rho	10	20	30	40	50	60	70	80	90	100
-0.9	70	40	30	25	25	20	20	20	15	15
-0.8	65	40	30	25	25	20	20	20	15	15
-0.7	60	40	30	25	25	20	20	20	15	15
-0.6	60	40	30	25	25	20	20	20	15	15
-0.5	55	40	30	25	25	20	20	20	15	15
-0.4	55	40	30	25	25	20	20	20	15	15
-0.3	55	40	30	25	25	20	20	20	20	15
-0.2	55	40	30	25	25	20	20	20	20	20
-0.1	50	40	30	25	25	25	20	20	20	20
0.0	50	40	30	30	25	25	20	20	20	20
0.1	50	40	35	30	25	25	25	20	20	20
0.2	50	40	35	30	30	25	25	25	20	20
0.3	50	40	35	30	30	25	25	25	25	20
0.4	50	40	35	35	30	30	25	25	25	25
0.5	50	45	40	35	35	35	30	30	30	25
0.6	50	45	40	40	40	35	35	30	30	30
0.7	50	45	45	45	45	40	40	35	35	35
0.8	50	50	50	50	50	45	45	40	40	40
0.9	45	50	50	50	50	50	50	45	45	45

Appendix Table 18

Minimum percent increase in post-intervention level to achieve >90% power at p<0.01 (treatment starts at 67% of the time series).

	Number of time periods									
rho	10	20	30	40	50	60	70	80	90	100
-0.9	75	35	30	25	20	20	20	15	15	15
-0.8	70	35	30	25	20	20	20	15	15	15
-0.7	60	35	30	25	20	20	20	15	15	15
-0.6	55	35	30	25	20	20	20	15	15	15
-0.5	50	35	30	25	20	20	20	15	15	15
-0.4	45	35	30	25	20	20	20	20	15	15
-0.3	45	35	30	25	20	20	20	20	15	15
-0.2	40	35	30	25	25	20	20	20	20	15
-0.1	40	35	30	25	25	20	20	20	20	20
0.0	40	35	30	25	25	25	20	20	20	20
0.1	40	35	30	25	25	25	20	20	20	20
0.2	40	35	30	30	30	25	25	20	20	20
0.3	40	35	35	30	30	25	25	25	25	20
0.4	40	40	35	35	30	30	30	25	25	25
0.5	40	40	35	35	35	30	30	30	25	25
0.6	40	45	40	40	35	35	35	30	30	30
0.7	40	45	40	40	40	40	40	35	35	35
0.8	40	45	45	45	45	45	45	40	40	40
0.9	40	45	50	50	50	50	50	45	45	45
